# Differential effects of emotional labor strategies on university counselors’ job burnout: the dual role of psychological capital as a mediator and a moderator

**DOI:** 10.3389/fpsyg.2026.1692256

**Published:** 2026-02-18

**Authors:** Yunbo Song, Honglin Yang

**Affiliations:** Faculty of Education, Yunnan Normal University, Kunming, China

**Keywords:** conservation of resources theory, deep acting, emotional labor strategies, job burnout, natural expression, psychological capital, surface acting, university counselor

## Abstract

**Background:**

University counselors are a high-risk group for job burnout, and how to alleviate their job burnout has become an increasingly urgent issue. Previous studies have paid less attention to the differential effects of emotional labor strategies on the job burnout of the counselors. This study aims to explore the association between various emotional labor strategies and job burnout among counselors, as well as the underlying mechanisms involving psychological capital as mediator and moderator.

**Methods:**

This study selected 747 counselors from six public universities in a western Chinese province using a cross-sectional design. Standardized scales were employed to measure emotional labor strategies, psychological capital, and job burnout. Data analysis was performed using the SPSS PROCESS macro (v4.3) to examine the dual role of psychological capital, separately testing its mediating effect in the relationship between emotional labor strategies and job burnout, and its moderating effect on their direct association.

**Results:**

The study results indicated that surface acting was significantly positively related to job burnout, whereas deep acting and natural expression were significantly negatively related to job burnout. This relationship was influenced by the mediating role of psychological capital, with effect sizes ranked as follows: deep acting > natural expression > surface acting. Furthermore, psychological capital moderated the relationships between surface acting/natural expression and job burnout.

**Conclusion:**

Results indicated that surface acting was significantly associated with job burnout, whereas deep acting and natural expression were negatively associated with job burnout. Psychological capital played a dual role: it not only mediated the relationship between emotional labor and job burnout, but also buffered the negative effect of surface acting while enhancing the protective effect of natural expression. Therefore, fostering psychological capital and promoting adaptive emotional labor strategies represent promising avenues for preventing job burnout among university counselors.

## 1 Introduction

In recent years, job burnout (hereafter, JB) has become an increasingly prominent social concern, with teachers being identified as a high-risk group receiving substantial scholarly attention (Shen et al., 2015; Feng et al., 2025). Current empirical research on teacher JB has primarily focused on primary/secondary school teachers and university faculty, while systematic investigations targeting university counselors (hereafter, UC) remain notably scarce (Pakdee et al., 2025; Liang and Yin, 2025), despite meta-analytic evidence indicating their high burnout risk (Kim and Ji, 2020). UC serve dual roles as both educators and administrators, functioning as core personnel for ideological and political education in higher education institutions. They represent crucial support for fulfilling the fundamental mission of fostering virtue through education, simultaneously performing triple roles as “administrators,” “educators,” and “service providers (Ding, 2021).” Their responsibilities encompass nine major functions including ideological guidance, psychological counseling, academic advising, and crisis intervention, with their JB levels significantly exceeding those of other professional groups (Feng, 2016). Similar to the high burnout risk observed among Chinese English teachers facing dual work demands (Zhang, 2022), UC’s multifaceted responsibilities and intense emotional labor further elevate their susceptibility to burnout. However, existing studies have predominantly examined role conflicts, work overload, or the enhancement of ideological education effectiveness among UC, while largely neglecting to explore the underlying mechanisms through which “emotional labor strategies(hereafter, ELS)”—a critical occupational characteristic—influence “JB” (Feng and Zhong, 2022). Although some studies have investigated the general relationship between emotional labor and burnout among service professionals (Hwang and Park, 2022; Peng et al., 2019), research specifically focusing on UC remains scarce, particularly regarding the differential effects of various ELS [surface acting (hereafter, SA), deep acting (hereafter, DA), natural expression (hereafter, NE)] and the potential mediating and moderating roles of psychological capital (hereafter, PsyCap) (Chen et al., 2021; Zhao et al., 2024). Furthermore, a study directly on Chinese university counselors has also examined the differential effects of emotional labor strategies on burnout (Li and Wang, 2020), yet the dual (mediating and moderating) role of psychological capital remains underexplored in an integrated model. Recent studies in other professions support these mechanisms; for instance, Hu and Wang (2022) found psychological capital mediated the relationship between deep acting and reduced burnout among service workers. This gap limits the understanding of how UC can effectively manage emotional demands to prevent burnout. JB, as a core issue in organizational behavior and occupational health research, not only impairs the physical and mental health of UC and reduces their work effectiveness—for example, job insecurity (a common stressor for UC) can trigger emotional exhaustion via rumination (Konkel and Heffernan, 2021)—but also adversely undermines student service quality and the achievement of higher education talent development goals. Particularly, UC frequently engage in emotion regulation during daily work (e.g., handling student conflicts and responding to emergencies), making their ELS (SA,DA,NE) potentially critical factors associated with JB. However, existing studies have not systematically examined the dual roles of “PsyCap” (e.g., self-efficacy, resilience, optimism) as both mediator and moderator in the relationship between ELS and JB. Grounded in Conservation of Resources theory (hereafter, COR theory), this study aims to address three core research questions regarding UC: What are the differential effects of SA, DA, and NE on JB? Does PsyCap mediate this relationship? Furthermore, does it also moderate it? Accordingly, the study pursues five specific objectives: to investigate the current status of JB; to assess its relationships with different ELS; to examine the mediating role of PsyCap; to explore its moderating effects; and ultimately, to provide a theoretical and practical foundation for designing targeted interventions. The findings are expected to contribute to building a high-quality counselor workforce and supporting the fundamental educational mission of fostering virtue.

## 2 Literature review and theoretical foundation

### 2.1 JB

Job burnout refers to a state of physical and mental exhaustion stemming from prolonged work stress, characterized by physical fatigue, psychological distress, and depletion of emotional resources (Maslach and Leiter, 2016). Scholars generally agree that JB comprises three core dimensions: emotional exhaustion (hereafter, EE), depersonalization (hereafter, DP), and reduced personal accomplishment (hereafter, RPA) (Maslach et al., 2001; Kinman, 2024). EE, the most representative diagnostic indicator, manifests as persistent energy depletion and emotional resource overdraft (Hu et al., 2017); DP reflects interpersonal dysfunction, featuring detachment and indifferent attitudes toward colleagues or service recipients (Demerouti et al., 2001); while RPA indicates diminished work efficacy, including lower self-evaluation, weakened sense of work purpose, and lack of achievement experience (Bakker et al., 2014). Recent research further highlights the heterogeneity of burnout experiences among human service professionals, identifying distinct profiles that call for differentiated interventions (Hao and Zhang, 2024). The COR theory proposed by Hobfoll (1989) serves as a key theoretical framework for explaining JB, positing that JB stems from dynamic imbalances between resource loss and gain. Resource loss constitutes a primary cause of JB—when facing work stress, individuals may experience continuous depletion of material (e.g., income), conditional (e.g., job stability), personal (e.g., self-efficacy), and energy (e.g., vitality) resources (Hobfoll et al., 2018; Yu et al., 2025). This loss spiral initiates defensive resource conservation, creating a vicious cycle of resource depletion that ultimately gives rise to EE and diminished coping capacity, explaining the positive correlation between resource loss and JB (Halbesleben et al., 2014; Lackey et al., 2025). Conversely, resource gain functions as a protective factor by providing stress buffers (e.g., social support), enhancing control (e.g., job autonomy), and generating resource accumulation effects (e.g., skill development), forming a “gain spiral” that typically correlates negatively with JB ( Amillano et al., 2024). Meta-analyses reveal that resource gain’s alleviating effect on JB significantly outweighs resource loss’s pathogenic impact (Alarcón et al., 2009). Hobfoll’s (2001) theoretical refinement notes that early COR theory overemphasized defensive responses to loss, whereas subsequent empirical studies demonstrate proactive resource investment strategies (e.g., PsyCap training, social network building) more effectively disrupt JB cycles (Dello and Stoykova, 2015; Wang et al., 2018).

### 2.2 ELS

Emotional labor has emerged as a distinct form of labor, holding equal importance with physical and mental labor. In service contexts, workers must not only expend physical and mental effort but also effectively manage their emotions (Gabriel et al., 2015). Hochschild (2012) first introduced the concept of “emotional labor,” revealing how flight attendants regulate emotional expressions to meet organizational demands. Subsequent research defines emotional labor as “the psychological process of aligning internal feelings with external expressions to comply with organizational display rules” (Grandey, 2000). When natural emotions conflict with job requirements, individuals engage DA or SA to adapt—a process termed ELS (Gabriel et al., 2015). The multidimensional strategies of emotional labor hold significant research value in organizational behavior. Diefendorff et al. (2005) established a tripartite framework comprising expression of NE, DA, and SA. NE refers to spontaneous emotional displays that align with organizational norms (Grandey and Melloy, 2017); DA involves cognitive reappraisal to achieve natural emotional congruence; whereas SA entails behavioral modulation of outward expressions despite internal dissonance. Modern research has advanced this understanding by identifying distinct profiles of emotional labor actors, demonstrating that individuals combine these strategies in nuanced ways rather than relying on a single approach (Gabriel et al., 2016). This distinction has been widely validated—for instance, SA correlates positively with EE (Plass and Kalyuga, 2019; Li and Wang, 2020). From the COR theory perspective, emotional labor constitutes a psychological resource depletion process (Hobfoll, 1989). Different ELS may thus differentially impact JB. Brotheridge and Lee (2002) found SA exacerbates DP and RPA, while DA mitigates these JB dimensions. Similarly, Grandey (2003) demonstrated SA increases EE, whereas DA’s reduction of cognitive dissonance buffers JB. Research specific to educational contexts reveals that NE has a protective effect against JB among teachers, a finding applicable to service professions. ELS yield dual effects: positive outcomes (e.g., service goal attainment, interpersonal harmony) enhance job satisfaction and organizational efficacy (Grandey, 2000), while negative consequences (e.g., EE) elevate absenteeism and turnover (Brotheridge and Lee, 2002). It is noteworthy that the relationship between ELS and outcomes can be complex; for example, in certain organizational climates (e.g., those perceived as dehumanizing), even DA may have ambivalent effects on employee wellbeing (Nguyen et al., 2022). Compared to other educators, UC face heightened emotional labor demands due to intensive affective investments in student interactions.

### 2.3 PsyCap

Psychological capital, as a higher-order construct, integrates four core dimensions: self-efficacy, hope, optimism, and resilience. Self-efficacy reflects an individual’s goal commitment and belief in success when facing challenges; hope represents goal-directed willpower and the ability to adjust pathways; optimism embodies positive expectations about present and future outcomes; while resilience refers to the dynamic process of rebounding from and adapting to adversity (Luthans et al., 2007a; Avey et al., 2011). Research indicates that PsyCap possesses state-like characteristics, demonstrating significantly greater malleability than stable personality traits (Luthans and Youssef, 2004; Luthans et al., 2008). From the perspective of COR theory, PsyCap functions as an internal cognitive resource buffer. By enhancing adaptive coping with emotional labor demands, it reduces the risk of resource depletion and consequently inhibits the onset of JB. Therefore, PsyCap is hypothesized to be negatively correlated with JB. A recent meta-analysis has consolidated this view, validating the robust negative association between PsyCap and various dimensions of JB (Orgambídez et al., 2025). Numerous studies have demonstrated that PsyCap, as a positive psychological resource, systematically shapes individuals’ attitudinal tendencies and behavioral decisions (Luthans et al., 2006; Avey et al., 2011; Newman et al., 2014). For instance, Avey et al.’s (2011) meta-analysis revealed that PsyCap not only positively predicts employees’ positive attitudes (including job satisfaction and organizational commitment) and behaviors (such as organizational citizenship behavior), but also enhances work performance. Conversely, it negatively predicts negative attitudes (e.g., turnover intention and work stress) and counterproductive behaviors (e.g., workplace deviance). Supporting this hypothesis, Luthans et al. (2007a) found that high PsyCap attenuates stress responses and effectively reduces turnover; Bakker and Demerouti (2017) showed that personal resources buffer the negative effects of job demands, reducing physical and mental fatigue while mediating the impact of job resources on JB. Critically, PsyCap is not merely a correlate but a developable resource. Evidence from intervention studies indicates that targeted training (e.g., cognitive-behavioral approaches) can effectively enhance PsyCap, which in turn improves emotional labor skills and reduces burnout risk (Guo and Chen, 2023).

### 2.4 The tripartite relationship: ELS, PsyCap, and JB

While existing studies have examined the individual effects of ELS and PsyCap on JB, few have adopted an integrated model to reveal their interactive relationships. Grounded in COR theory, resource loss and resource gain exert both independent and synergistic effects on JB (Hobfoll et al., 2020). Research suggests PsyCap may play dual roles in the relationship between ELS and JB : (1) as a mediator, where different ELS influence JB through distinct patterns of resource depletion—NE (the most adaptive strategy) incurs minimal psychological resource consumption (Gabriel et al., 2015), DA requires moderate resource investment for natural emotional alignment (Grandey and Melloy, 2017), while SA generates substantial depletion due to cognitive dissonance, a mediating pathway that has garnered recent empirical support in adjacent caregiving professions (Lee and Kim, 2020); and (2) as a moderator, where higher PsyCap enables cognitive reappraisal to reframe emotional labor stressors (Luthans et al., 2015; Gross, 2015)—a mechanism consistent with Zeng et al.’s (2020) finding that emotional regulation self-efficacy strengthens the protective effect of organizational support against burnout. These findings collectively establish a theoretical foundation for understanding PsyCap’s dual-pathway mechanisms (mediation and moderation) linking ELS to JB (Chen et al., 2021). Notably, recent empirical work on college teachers has verified that psychological capital significantly moderates the effects of emotional labor strategies on job burnout (Yin, 2023), providing direct support for the moderating hypothesis in an educational context.

## 3 Research hypotheses and conceptual framework

### 3.1 Research hypotheses

Based on the literature review and COR theory, the following hypotheses are proposed:

H1: Emotional labor strategies differentially affect job burnout among university counselors. Specifically, surface acting is positively associated with job burnout, whereas deep acting and natural expression are negatively associated with job burnout.

H2: Psychological capital is negatively associated with job burnout.

H3: Psychological capital mediates the relationship between emotional labor strategies and job burnout.

H4: Psychological capital moderates the relationship between emotional labor strategies and job burnout.

### 3.2 Conceptual framework

**FIGURE 1:**
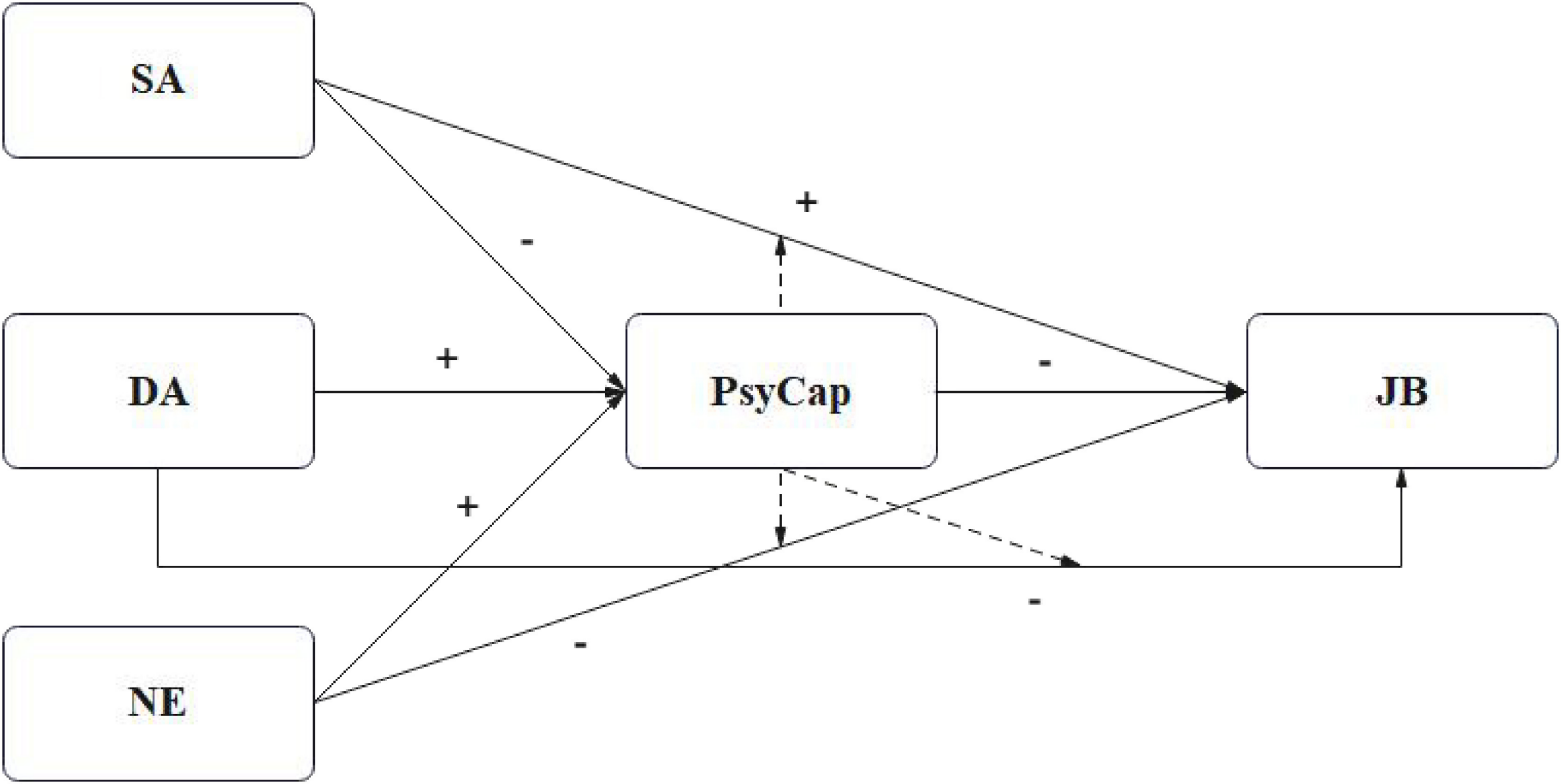
Conceptual framework of the study. SA, surface acting; DA, deep acting; NE, nature expression; PsyCap, psychological capital; JB, job burnout. The hypothesized positive (+) and negative (–) associations are indicated on the paths. The dashed line from PsyCap to the path between ELS and JB represents the moderating effect.

Drawing on the hypotheses grounded in COR theory, the conceptual framework of this study is visually presented in Figure 1. The model illustrates the proposed dual role of PsyCap: (a) as a mediator that explains the mechanism through which ELS influence JB, and (b) as a moderator that alters the strength of the direct relationships between ELS and JB.

## 4 Materials and methods

### 4.1 Participants and procedure

This cross-sectional study employed a convenience sampling method to recruit 747 full-time UC from six public undergraduate institutions in a southwestern Chinese province in 2025. Data were collected using a standardized UC JB Survey Questionnaire, which included three validated scales: the ELS Scale, the JB Scale, and the PsyCap Scale, along with a demographic questionnaire. To enhance response validity, participants received CNY 35 worth of cultural creative products as compensation, yielding 744 valid questionnaires (effective response rate: 99.7%). All data were independently double-entered into SPSS 27.0, with 100% accuracy validated through consistency checks. The raw data supporting this study are available in Supplementary Table 1.

The study was approved by the Academic Ethics Committee of the School of Applied Technology, Yunnan Minzu University (Approval No. YMU-AEC2025-003). The protocol strictly adhered to the ethical principles of the Declaration of Helsinki, China’s “Ethical Review Measures for Biomedical Research Involving Humans,” and relevant Ministry of Education regulations. Specific ethical safeguards included: anonymized data processing, obtaining written informed consent from all participants, and implementing voluntary participation with an opt-out mechanism at any time to ensure full compliance with ethical standards.

### 4.2 Measurements

#### 4.2.1 ELS

The ELS Scale developed by Diefendorff et al. (2005) was culturally adapted to measure UC ELS, comprising 14 items across three dimensions: SA (7 items) assessing behavioral regulation of emotional displays, DA (4 items) evaluating internal emotional adjustment, and NE (3 items) reflecting natural emotional responses. Responses were recorded on a five-point Likert scale (1 = “strongly disagree” to 5 = “strongly agree”), with dimension scores calculated as means (higher scores indicating more frequent strategy use). The scale demonstrated good reliability in this study (Cronbach’s α = 0.828).

#### 4.2.2 JB

The study employed the adapted Maslach JB Inventory-Educators Survey for UC (MBI-ES; Maslach and Jackson, 1996), consisting of 22 items across three dimensions: EE (9 items), DP (5 items), and RPA (8 items). Responses were recorded on a seven-point Likert scale (0 = “never” to 6 = “daily”), with raw scores calculated by summing EE (range: 0–54) and DP (range: 0–30) items, while personal accomplishment items were reverse-scored before summation (range: 0–48). Following the manual’s criteria (Li and Shi, 2003), cutoff scores indicating high JB were: ≥27 for EE, ≥10 for DP, and ≤18 for RPA. The scale demonstrated excellent reliability (Cronbach’s α = 0.888), consistent with both the original English version and Chinese adaptation.

#### 4.2.3 PsyCap

The study utilized the culturally adapted PsyCap Questionnaire (PCQ-24; Luthans et al., 2007b) to assess UC PsyCap, comprising 24 items across four dimensions: self-efficacy (6 items), optimism (6 items), resilience (6 items), and hope (6 items). Responses were collected using a six-point Likert scale (1 = “strongly disagree” to 6 = “strongly agree”), with total scores calculated by summing all items (after reverse-scoring specified items) - higher scores indicating greater PsyCap. The scale demonstrated excellent internal consistency in the current study (Cronbach’s α = 0.902).

### 4.3 Preliminary data analysis and assumption testing

Prior to hypothesis testing, the suitability of the data for parametric analyses was assessed. The core variables showed acceptable descriptive statistics: SA (*M* = 2.54, SD = 0.98), DA (*M* = 3.48, SD = 0.91), NE (*M* = 3.70, SD = 0.70), JB (*M* = 1.95, SD = 0.96), and PsyCap (*M* = 4.52, SD = 0.69). Their skewness (absolute values: 0.12–0.93) and kurtosis (absolute values: 0.42–2.26) fell within acceptable ranges (e.g., below |2| and |7|, respectively), indicating no severe non-normality. Although Shapiro-Wilk tests were significant (*p* < 0.001)—a common outcome in large samples (*N* = 744) due to high test sensitivity—the observed distribution shapes, combined with the large sample size (which ensures robustness via the Central Limit Theorem) and visual checks verifying no extreme outliers, supported the use of parametric regression-based analyses as planned. Diagnostic checks on the regression models (e.g., residual plots) further verified that the key assumptions of independence, normality, and homoscedasticity were reasonably met.

### 4.4 Statistical analysis procedures for hypothesis testing

**FIGURE 2:**
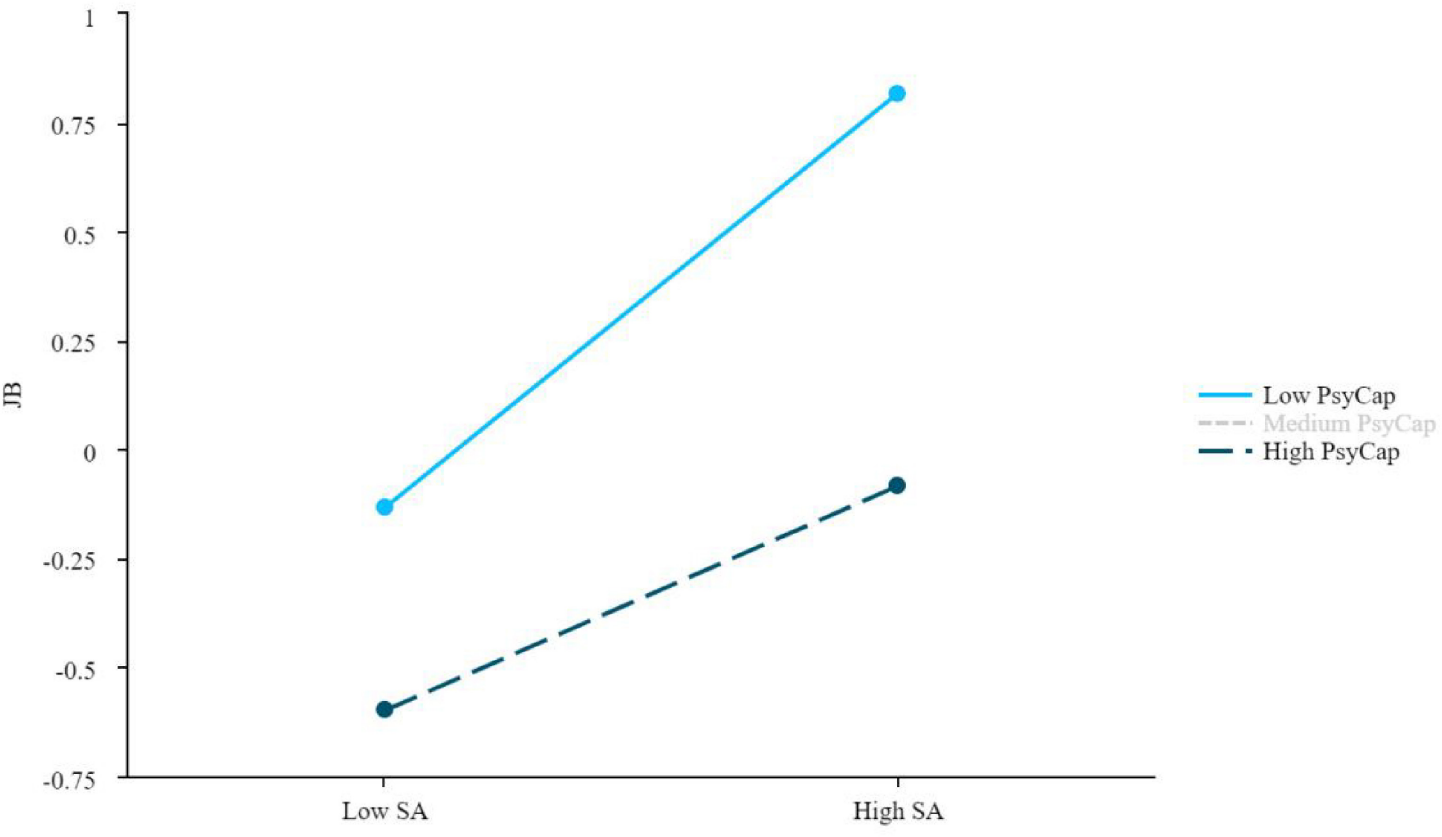
Moderating effect of PsyCap between SA and JB. JB, job burnout; SA, surface acting; PsyCap, psychological capital.

**FIGURE 3:**
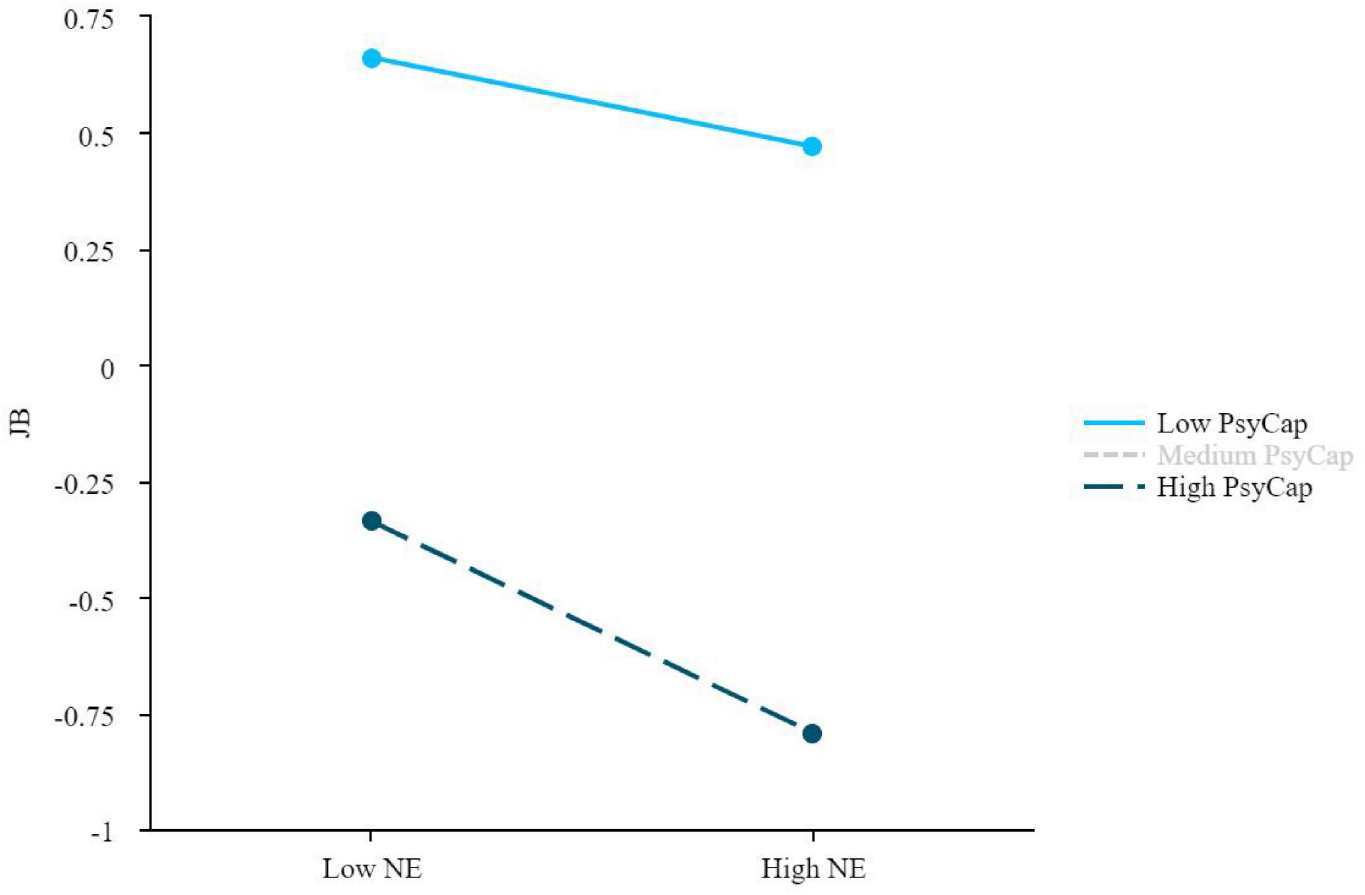
Moderating effect of PsyCap between NE and JB. JB, job burnout; NE, nature expression; PsyCap, psychological capital.

Data analysis was conducted using SPSS 27.0 with the PROCESS 4.3 macro, following a sequential analytical approach. First, preliminary data checks confirmed the suitability of the data for parametric analyses (as reported in see section “4.3 Preliminary data analysis and assumption testing”), while Harman’s single-factor test examined common method bias (Results 5.1). Descriptive statistics summarized participant characteristics and job burnout prevalence (Results 5.2–5.3), and one-way ANOVAs identified demographic covariates (*p* < 0.05) for subsequent models (Results 5.4). Second, Pearson correlations examined the bivariate relationships among core variables (in their original metric), providing initial evidence for H1 and H2 (Results 5.5). Third, to ensure comparability of effect sizes across scales with different metrics (five-point for ELS, seven-point for JB, six-point for PsyCap), all variables were standardized (converted to z-scores) prior to conducting the formal hypothesis tests. To test H3, PROCESS Model 4 was then employed in three separate mediation analyses examining whether PsyCap mediated the relationships between each emotional labor strategy and JB (Results 5.6). Fourth, to test H4, PROCESS Model 1 was used in three separate moderation analyses testing whether PsyCap moderated these direct relationships (Results 5.7). For the moderation analyses, the independent variables and moderator were mean-centered before creating the interaction terms to reduce multicollinearity. Furthermore, to visually clarify the nature of the significant moderation effects, simple slope plots were generated depicting the relationship between each emotional labor strategy and JB at high (+1 SD) and low (−1 SD) levels of PsyCap (see Figures 2, 3 in section “5.7 Test of the moderate effect of PsyCap on the relationship between ELS and JB”). The significance of indirect effects (H3) and conditional effects (H4) was assessed using the bias-corrected bootstrap method with 5,000 resamples (95% CI not including zero indicated significance).

## 5 Results

### 5.1 Common methods bias

Given that all data were collected through self-report measures, potential common method bias (CMB) was assessed using Harman’s single-factor test prior to statistical analyses. Exploratory factor analysis revealed that the first common factor accounted for 27.248% of the total variance, substantially below the 40% threshold (Podsakoff et al., 2003), indicating no significant common method bias in this study and supporting the validity of subsequent analyses.

### 5.2 Participant characteristics

The study sample comprised 744 UC, with a gender distribution skewed toward females (55.6% vs. 44.4%). The majority were married (69%), while 30.6% were unmarried, and a negligible proportion (0.4%) reported other marital statuses. In terms of professional rank, junior titles constituted the largest group (44.4%), followed by intermediate (35.5%) and associate senior titles (11.3%); only 1.2% held full senior titles, and 7.6% had undetermined ranks. Regarding administrative roles, 35% held no administrative position, with clerks (31%) and section chiefs (14.9%) being the most common roles; deputy division-level positions and above collectively accounted for 18.1%. The distribution of years dedicated to full-time counseling exhibited a pronounced right skew: 63.3% had 1–5 years of experience, 25% had 6–10 years, and 11.7% had 11 years or more. Weekly hours devoted to student-related work revealed that 64.5% spent 41 h or more—including 34.6% exceeding 50 h—while 15.7% worked 31–40 h, and 19.8% worked 30 h or less.

### 5.3 Prevalence of JB

Among all respondents, UC reported varying degrees of JB across dimensions. EE showed the highest prevalence, with 68.1% exhibiting moderate-to-severe levels; RPA followed closely at 67.7%; while DP demonstrated relatively lower rates, with 40% reaching moderate-to-severe thresholds (see Table 1 for detailed results).

**TABLE 1:** Job burnout (JB) and detection of various dimensions among UC.

JB level	EE		DP		RPA	
	Frequency	Rate	Frequency	Rate	Frequency	Rate
Mild JB	237	31.9%	446	60%	240	32.3%
Moderate JB	263	35.4%	223	30%	288	38.7%
Severe JB	244	32.7%	75	10%	216	29.0%

### 5.4 Demographic differences in UC JB levels

One-way ANOVA revealed significant differences in JB levels across demographic groups (see Table 2). Specifically: female UC reported significantly higher JB (*M* = 49.856) than males (*M* = 45.000); married participants showed greater JB (*M* = 49.455) than unmarried counterparts (*M* = 44.092); those with 6–10 years of experience exhibited the most severe JB (*M* = 50.993); and UC working ≤20 h/week demonstrated the highest JB levels (*M* = 52.535), with significant decreases as work hours increased. Furthermore, JB showed a stepwise escalation from junior (*M* = 45.065) to senior positions (*M* = 54.143).

**TABLE 2:** Comparison of job burnout (JB) levels among UC based on different demographic information.

Variables	Categories	JB score	(F/t)	*P*
Gender	Male	45	−2.455	0.014*
	Female	49.856	–	–
Marital status	Married	49.455	6.483	0.002**
	Unmarried	44.092	–	–
	Other	7	–	–
Professional title	Junior	45.065	1.693	0.15
	Intermediate	48.969	–	–
	Associate senior	51.575	–	–
	Senior	54.143	–	–
	Undetermined	49.46	–	–
Work experience (years)	1–5	46.343	3.409	0.017*
	6–10	50.993	–	–
	11–15	49.681	–	–
	16–20	16.333	–	–
Weekly working hours	≤20	52.535	4.6	0.001***
	21–30	52.313	–	–
	31–40	52.515	–	–
	41–50	47.339	–	–
	≥51	42.874	–	–

*P < 0.05;

** < 0.01;

*** < 0.001.

### 5.5 Descriptive analysis and correlation test of core variables

Descriptive statistics and bivariate correlations for all core variables are summarized in Table 3. The analysis yielded a clear and statistically significant pattern of associations, providing strong initial support for the proposed hypotheses. In support of H1, SA was positively correlated with all three dimensions of JB (EE, DP, and RPA), whereas both DA and NE were negatively correlated with them, demonstrating the differential associations of ELS with JB. Furthermore, H2 was strongly supported as PsyCap and its four constituent dimensions (self-efficacy, optimism, resilience, and hope) all showed significant negative correlations with JB. Notably, the correlation matrix also established the preliminary linkages necessary for H3 (the mediation hypothesis): both adaptive strategies (DA and NE) were positively correlated with PsyCap, which was in turn negatively correlated with JB. Conversely, SA was negatively correlated with PsyCap.

**TABLE 3:** Bivariate correlation among all observed variables.

Variable	1	2	3	4	5	6	7	8	9	10
1. SA	1	–	–	–	–	–	–	–	–	–
2. DA	−0.342**	1	–	–	–	–	–	–	–	–
3. NE	−0.435**	0.374**	1	–	–	–	–	–	–	–
4. Self-efficacy	−0.524**	0.360**	0.455**	1	–	–	–	–	–	–
5. Hope	−0.546**	0.360**	0.443**	0.575**	1	–	–	–	–	–
6. Resiliency	−0.548**	0.341**	0.456**	0.548**	0.644**	1	–	–	–	–
7. Optimism	−0.522**	0.353**	0.497**	0.545**	0.620**	0.634**	1	–	–	–
8. EE	0.539**	−0.193**	−0.350**	−0.411**	−0.434**	−0.429**	−0.388**	1	–	–
9. DP	0.483**	−0.236**	−0.346**	−0.392**	−0.422**	−0.422**	−0.415**	0.600**	1	–
10. RPA	0.536**	−0.251**	−0.369**	−0.431**	−0.428**	−0.442**	−0.441**	0.527**	0.622**	1
Mean	2.544	3.482	3.773	4.923	4.455	4.472	4.214	2.363	1.161	1.982
SD	0.977	0.908	0.845	0.682	0.89	0.79	0.801	1.409	4.245	1.272

*P < 0.05;

** < 0.01.

### 5.6 Tests of mediating effects of PsyCap

To test H3, which posits that the relationship between ELS and JB is mediated by PsyCap, we performed three-step regression analyses for SA, DA, and NE.

#### 5.6.1 Test of the mediate effect of PsyCap on the relationship between SA and JB

For SA (Table 4), the first step verified its significant positive association with JB (β = 0.622, *p* < 0.001). The second step revealed that SA reduced PsyCap (β = −0.619, *p* < 0.001), indicating resource depletion. When both were included (Step 3), SA (β = 0.397, *p* < 0.001) and PsyCap (β = −0.362, *p* < 0.001) remained significant, with PsyCap mediating 36.01% of the total effect (indirect effect: 0.224). This partial mediation effect is consistent with H3, supporting a dual-pathway model: SA was directly associated with higher JB and indirectly linked to it through lower PsyCap.

**TABLE 4:** Mediation model test for SA-PsyCap-JB.

Regression equation			Overall fit indicators			Significance of regression coefficients			
Step	Result variable	Predictor variable	R	R^2^	F	β	SE	*t*	95% CI
Step 1	JB	SA	0.637	0.405	59.552***	0.622	0.033	18.961	[0.557, 0.686]
Step 2	PsyCap	SA	0.646	0.417	62.553***	−0.619	0.030	−20.536	[−0.679, −0.560]
Step 3	JB	SA	0.686	0.471	68.036***	0.397	0.040	9.884	[0.318, 0.476]
		PsyCap	–	–	–	−0.362	0.041	−8.729	[−0.443, −0.281]
**Total, direct, and indirect effects of X on Y**									
**Effect decomposition**						**β**	**SE**	***t***	**95% CI**
Total effect	–	–	–	–	–	0.622	0.033	18.961	[0.557, 0.686]
Direct effect	–	–	–	–	–	0.397	0.040	9.884	[0.318, 0.476]
Indirect effect	–	–	–	–	–	0.224	0.039	–	[0.152, 0.307]

Covariate data etc., are omitted in the table; ***p < 0.001.

#### 5.6.2 Test of the mediate effect of PsyCap on the relationship between DA and JB

For DA (Table 5), regression analyses revealed: (1) a significant negative association with JB (β = −0.293, *p* < 0.001); (2) a positive effect on PsyCap (β = 0.463, *p* < 0.001); and (3) complete mediation through PsyCap (β = −0.622, *p* < 0.001), accounting for 98.29% of the total effect (indirect effect: 0.288). The direct effect became non-significant when accounting for PsyCap (β = −0.006, *p* > 0.05), demonstrating an near-complete mediation effect, which provides strong support for H3. This shows that DA’s protective effect operates almost entirely by enhancing psychological resources, unlike SA’s partial mediation pattern.

**TABLE 5:** Mediation model test for DA-PsyCap-JB.

Regression equation			Overall fit indicators			Significance of regression coefficients			
Step	Result variable	Predictor variable	R	R^2^	F	β	SE	*t*	95% CI
Step 1	JB	DA	0.34	0.115	11.406***	−0.293	0.046	−6.425	[−0.383, −0.204]
Step 2	PsyCap	DA	0.433	0.187	20.134***	0.463	0.041	11.373	[0.383, 0.543]
Step 3	JB	DA	0.622	0.387	48.132***	−0.006	0.042	−0.140	[−0.088, 0.076]
		PsyCap	–	–	–	−0.622	0.038	−16.435	[−0.696, −0.547]
**Total, direct, and indirect effects of X on Y**									
**Effect decomposition**						β	SE	*t*	95% CI
Total effect	–	–	–	–	–	−0.293	0.046	−6.425	[−0.383, −0.204]
Direct effect	–	–	–	–	–	−0.006	0.042	−0.14	[−0.088, 0.076]
Indirect effect	–	–	–	–	–	−0.288	0.039	–	[−0.364, −0.210]

Covariate data etc., are omitted in the table; ***p < 0.001.

#### 5.6.3 Test of the mediate effect of PsyCap on the relationship between NE and JB

For NE (Table 6), three-step regression analyses revealed: (1) a significant negative association with JB (β = −0.454, *p* < 0.001), demonstrating NE’s protective effect; (2) positive effects on PsyCap (β = 0.566, *p* < 0.001), demonstrating its resource-enhancing capacity; and (3) partial mediation through PsyCap (indirect effect = 0.307, 67.62% of total effect) when both variables predicted JB (NE: β = −0.147, *p* < 0.001; PsyCap: β = −0.543, *p* < 0.001). This significant partial mediation again provides clear evidence for H3. This mediation strength positions NE between SA (36.01%) and DA (98.29%), indicating dual protective pathways - direct emotional regulation benefits coupled with stronger indirect effects via PsyCap augmentation.

**TABLE 6:** Mediation model test for NE-PsyCap-JB.

Regression equation			Overall fit indicators			Significance of regression coefficients			
Step	Result variable	Predictor variable	R	R^2^	F	β	SE	*t*	95% CI
Step 1	JB	NE	0.475	0.226	25.475***	−0.454	0.039	−11.588	[−0.531, −0.377]
Step 2	PsyCap	NE	0.566	0.321	41.263***	0.566	0.034	16.582	[0.499, 0.633]
Step 3	JB	NE	0.632	0.399	50.665***	−0.147	0.042	−3.529	[−0.229, −0.065]
		PsyCap	–	–	–	−0.543	0.041	−13.267	[−0.624, −0.463]
**Total, direct, and indirect effects of X on Y**									
**Effect decomposition**						**β**	**SE**	***t***	**95% CI**
Total effect	–	–	–	–	–	−0.454	0.039	−11.588	[−0.531, −0.377]
Direct effect	–	–	–	–	–	−0.147	0.042	−3.529	[−0.229, −0.065]
Indirect effect	–	–	–	–	–	−0.307	0.035	–	[−0.377, −0.240]

Covariate data etc., are omitted in the table; ***p < 0.001.

Comparative mediation analysis revealed distinct patterns: DA showed complete mediation (98.29%) through PsyCap, NE demonstrated partial mediation (67.62%) with dual pathways, while SA exhibited both direct effects and weaker partial mediation (36.01%). Crucially, these results collectively show that H3 receives data support, revealing PsyCap as a significant mediating mechanism for all three ELS, albeit to varying degrees.

### 5.7 Test of the moderate effect of PsyCap on the relationship between ELS and JB

This study investigated the moderating role of PsyCap in the relationships between three ELS and JB to directly test H4. Moderation analyses revealed significant differential patterns across strategies (all *p*s < 0.05).

#### 5.7.1 Test of the moderate effect of PsyCap on the relationship between SA and JB

As shown in Table 7, PsyCap demonstrated a significant buffering effect on the SA-JB relationship (interaction β = −0.109, 95% CI [−0.165, −0.054], *p* < 0.05). Conditional effect analysis showed that SA’s positive association with JB was stronger at low PsyCap levels (−1 SD: β = 0.476, 95% CI [0.388, 0.564]) compared to high levels (+1 SD: β = 0.259, 95% CI [0.154, 0.364]), representing a 45.6% attenuation. This significant buffering effect provides strong and clear evidence supporting H4, indicating that PsyCap mitigates JB by offsetting the resource depletion associated with SA.

**TABLE 7:** Moderation effect of PsyCap × SA on JB.

Y: JB			
	β	SE	95% CI
X: SA	0.367	0.041	[0.288, 0.447]
W: PsyCap	−0.342	0.041	[−0.423, −0.261]
X × W	−0.109	0.028	[−0.165, −0.054]
**Conditional indirect effect of X on Y of the moderator**			
−1 SD	0.476	0.045	[0.388, 0.564]
Mean	0.367	0.041	[0.288, 0.447]
+1 SD	0.259	0.053	[0.154, 0.364]

Covariate data etc., are omitted in the table.

The significant moderating pattern is visually illustrated in Figure 2. A clear divergence is observed in the slope representing the association between SA and JB for UC with high versus low PsyCap. The steeper positive slope under low PsyCap conditions indicates a stronger positive SA-JB association. Conversely, the attenuated slope under high PsyCap conditions provides a graphical representation of the moderating role of PsyCap, which is associated with a weaker positive relationship between SA and JB among UC.

#### 5.7.2 Test of the moderate effect of PsyCap on the relationship between NE and JB

Table 8 presents the moderating role of PsyCap in the association between NE and JB, revealing a significant negative interaction (β = −0.066, 95% CI [−0.125, −0.008], *p* < 0.05) that indicates PsyCap enhances NE’s protective effect. Conditional effects analysis showed that while NE had a moderate negative association with JB at low PsyCap levels (−1 SD: β = −0.096, 95% CI [−0.189, −0.002]), this protective effect strengthened substantially at high levels (+1 SD: β = −0.227, 95% CI [−0.336, −0.119]), representing a 136% increase in effect size. This significant enhancing effect offers further support for H4, demonstrating a synergistic relationship where PsyCap amplifies the resource-gaining properties of NE.

**TABLE 8:** Moderation effect of PsyCap × NE on JB.

Y: JB			
	β	SE	95% CI
X: NE	−0.162	0.042	[−0.244, −0.079]
W: PsyCap	−0.566	0.042	[−0.649, −0.484]
X × W	−0.066	0.030	[−0.125, −0.008]
**Conditional indirect effect of X on Y of the moderator**			
−1 SD	−0.096	0.047	[−0.189, −0.002]
Mean	−0.162	0.042	[−0.244, −0.079]
+1 SD	−0.227	0.055	[−0.336, −0.119]

Covariate data etc., are omitted in the table.

Figure 3 visually presents the moderating effect of PsyCap on the NE-JB relationship. Both slopes are negative, consistent with the overall negative association between NE and JB. Notably, the slope for UC with high PsyCap is markedly steeper, indicating that the negative NE-JB relationship is stronger when coupled with higher psychological resources. This pattern provides graphical support for the significant interaction, wherein higher PsyCap is linked to a strengthened protective association between NE and JB among UC.

#### 5.7.3 Test of the moderate effect of PsyCap on the relationship between DA and JB

Table 9 presents the moderating effect of PsyCap on the relationship between DA and JB. The analysis revealed a marginally significant negative interaction (β = −0.051, 95% CI [−0.109, −0.007], *p* = 0.08), suggesting PsyCap may weakly enhance DA’s effects on JB. Conditional effect analysis showed that at high PsyCap levels (+1 SD), DA exhibited a trend toward reducing JB (β = −0.073, 95% CI [−0.185, 0.039]), though this effect was not statistically significant. Given the marginal significance and confidence intervals including zero for conditional effects, this result does not provide robust evidence for H4 in the context of DA, indicating potential moderation that requires further verification with larger samples.

**TABLE 9:** Moderation effect of PsyCap × DA on JB.

Y: JB			
	β	SE	95% CI
X: DA	−0.022	0.043	[−0.107, 0.062]
W: PsyCap	−0.635	0.039	[−0.711, −0.559]
X × W	−0.051	0.03	[−0.109, −0.007]
**Conditional indirect effect of X on Y of the moderator**			
−1 SD	0.028	0.046	[−0.062, 0.119]
Mean	−0.022	0.043	[−0.107, 0.062]
+1 SD	−0.073	0.057	[−0.185, 0.039]

Covariate data etc., are omitted in the table.

Comparative analysis of the three strategies revealed a gradient in PsyCap’s moderating strength: SA (β = −0.109) > NE (β = −0.066) > DA (β = −0.051). Collectively, these results offer partial support for H4, indicating a significant moderating role of PsyCap for SA and NE, but not for DA. This provides critical evidence for targeted intervention development.

## 6 Discussion

### 6.1 Critical analysis of research results

This study provides evidence that ELS differentially influence JB among UC, and that PsyCap plays a dual role as both a mediator and a moderator in these relationships. These observed patterns offer several critical insights worthy of deeper analysis.

First, the distinct directional associations of ELS with JB—positive for SA and negative for DA and NE—are consistent with COR theory’s prediction (Hobfoll, 1989) that emotional labor can be either resource-depleting or resource-conserving. SA, characterized by emotional dissonance, shows association with a “loss spiral” through the depletion of psychological resources (Zapf, 2002). In contrast, DA and NE, by aligning internal emotions with external expressions, may be linked to resource conservation or generation (Goldberg and Grandey, 2007), thereby correlating with lower burnout levels. This finding appears particularly relevant for UC, whose work involves frequent and intense emotional interactions, suggesting that the choice of emotional strategy represents an important correlate of occupational wellbeing.

Second, the mediation analyses reveal nuanced pathways through which ELS are associated with JB. The near-complete mediation of DA through PsyCap indicates that DA’s protective effect may operate primarily by enhancing psychological resources—a pattern consistent with cognitive reappraisal mechanisms (Grandey, 2003) and complementary to Yin et al.’s (2022) finding that positive coping styles mediate the sleep quality-burnout relationship among healthcare professionals. In contrast, the partial mediation observed for SA and NE suggests that these strategies also exert direct effects on burnout beyond PsyCap. For SA, this direct path may represent the immediate cognitive and emotional costs of faking emotions; for NE, it may reflect the inherent benefits of spontaneous, congruent emotional expression. This differential mediation pattern indicates that not all emotional labor operates through the same psychological mechanisms (Diefendorff et al., 2005).

Third, the moderation results highlight the conditional nature of these relationships. PsyCap significantly moderated the detrimental association of SA with JB and strengthened the protective relationship between NE and JB, but did not moderate the DA–JB link. These patterns suggest that PsyCap’s role as a personal resource may be most salient in contexts of high emotional dissonance (SA) or high authenticity (NE). Similar to Li et al.’s (2024) finding that cognitive reappraisal (a core emotional regulation strategy) moderates the depression-burnout link, the present findings demonstrates that personal psychological resources can alter the strength of stressor-burnout relationships (Liu et al., 2024). The non-significant moderation for DA implies that DA’s effectiveness could depend more on skill-based or situational factors—a finding that warrants further theoretical refinement regarding the boundary conditions of PsyCap’s moderating role (Chen et al., 2021; Zhao et al., 2024). For instance, the high level of role conflict inherent in UC’s work (Zhou and Li, 2021) may create a situational constraint that diminishes the potential buffering effect of PsyCap on the DA-JB relationship.

### 6.2 Comparison with related studies

This study’s results align with and extend the existing literature on emotional labor, burnout, and psychological resources. Regarding ELS, the observed patterns corroborate prior research in service professions showing that SA is positively associated with burnout, whereas DA and NE are negatively related to it (Brotheridge and Grandey, 2002; Grandey, 2003). Notably, Liang and Yin (2025) recently revealed these relationships in a large sample of 488 Chinese university counselors, further validating the generalizability of emotional labor theory to this specific group. However, much of this research has focused on frontline service workers, nurses, or teachers, whereas the current study examines UC—a hybrid professional group that blends educational, administrative, and emotional support roles. This focus extends the generalizability of emotional labor theory to an understudied population. The high prevalence of burnout observed among UC in this sample further underscores the need for targeted interventions in this group.

Concerning psychological capital, the mediation findings are consistent with studies highlighting PsyCap as a key mediator between job demands and wellbeing outcomes (Avey et al., 2011; Luthans et al., 2007a; Hu and Wang, 2022). However, previous research has seldom examined its mediating role across different emotional labor strategies within a single integrated model. The present study advances this line of inquiry by showing that PsyCap mediates all three strategies but to varying degrees—exhibiting near-complete mediation for DA and partial mediation for SA and NE. This nuanced pattern provides a more granular understanding of how personal resources interact with emotion regulation processes.

In terms of moderation, the buffering effect of PsyCap on the SA–job burnout link aligns with prior research indicating that personal resources mitigate the impact of job demands (Xanthopoulou et al., 2007). However, the enhancing effect of PsyCap on NE’s protective role represents a novel contribution, suggesting that psychological resources may not only weaken negative pathways but also strengthen positive ones. The non-significant moderating effect for DA contrasts with some earlier findings (e.g., Chen et al., 2021), which may be attributed to differences in sample characteristics or operationalization of DA. This discrepancy highlights the need for further research to clarify the boundary conditions of PsyCap’s moderating role.

Theoretical integration with COR theory constitutes an additional contribution. While prior studies have used COR theory to explain burnout (Hobfoll, 1989), few have systematically applied it to differentiate the resource dynamics of specific emotional labor strategies. This study addresses this gap by framing SA as a resource loss mechanism, DA as resource investment, and NE as resource conservation—a conceptualization that enriches both the emotional labor and COR literatures (Hobfoll et al., 2018; Chen et al., 2021).

### 6.3 Connection between theoretical significance and practical implications

The theoretical insights from this study translate into actionable recommendations for preventing burnout among UC. These insights are grounded in the observed associations between ELS, PsyCap, and JB, reinforcing the importance of integrating emotion regulation and positive psychological resource frameworks at the theoretical level. The patterns observed suggest that burnout interventions should target not only the reduction of maladaptive strategies (like SA) but also the cultivation of adaptive ones (like DA and NE) and the enhancement of personal resources (like PsyCap). This approach aligns with COR theory’s emphasis on preventing resource loss while promoting resource gain.

Based on the empirical relationships identified, a dual-pathway intervention model comprising three complementary components is supported: (1) strategy-focused training to help UC recognize the costs of SA and develop skills in DA and NE, as suggested in prior training studies (Liu et al., 2008); (2) PsyCap development initiatives that leverage its state-like nature through structured interventions (Luthans et al., 2008; Avey et al., 2010; Luthans and Youssef-Morgan, 2017), integrating workshops on self-efficacy, optimism, hope, and resilience into UC professional development; recent intervention studies have demonstrated the effectiveness of such approaches in this specific population (Guo and Chen, 2023); (3) Optimization of Human Resource Management (HRM) practices. Beyond individual-focused training, universities should review and improve HRM systems (e.g., performance evaluation, reward mechanisms) that may inadvertently encourage maladaptive emotional labor. Supportive and fair HRM practices have been shown to foster positive work attitudes and behaviors (Huang and He, 2022), which could indirectly reduce reliance on surface acting and (4) organizational support structures, such as reduced caseloads, regular supervision, and a culture that values authentic emotional expression (Feng and Zhong, 2022).

The moderation analyses further indicate that interventions could be tailored to individual differences in PsyCap. For counselors low in PsyCap, priority might be given to reducing SA and building basic psychological resources; for those already high in PsyCap, fostering NE could yield amplified benefits. This personalized approach aligns with contemporary trends toward precision prevention in occupational health (Chen et al., 2021). Taken together, this study contributes to theoretical understanding of how ELS and psychological resources interact to influence burnout, offering actionable insights for supporting UC wellbeing and professional effectiveness.

### 6.4 Limitations and future research directions

Despite its contributions, this study has several limitations. First and foremost, the cross-sectional design precludes definitive causal inferences. While the mediation and moderation analyses align with theoretical causal processes, the observed relationships must be interpreted as coexisting statistical patterns rather than established cause-and-effect sequences. The terms “mediation,” “moderation,” “predict,” and “protective/risk factor” are used in a statistical and theoretical sense to describe the structure of relationships among variables, as is common in cross-sectional research using such models (Hayes, 2018). Future longitudinal or experimental research is needed to verify the suggested temporal and directional patterns. Second, the sample’s geographic concentration in one Chinese region may limit generalizability, warranting replication in more diverse cultural and institutional contexts. Third, reliance solely on self-report measures introduces the possibility of common method variance inflating relationships; incorporating multi-source assessments or objective indicators would strengthen future findings. Finally, the non-significant moderating role of PsyCap in the DA–JB relationship merits further investigation, potentially examining other moderators like organizational support or client characteristics.

## 7 Conclusion

Guided by COR theory, this study delineates the differential impacts of ELS on JB among UC and, more importantly, unveils the dual role of PsyCap as both a mediating mechanism and a protective moderator. Findings indicate that SA is positively associated with JB, whereas DA and NE are negatively associated with JB. Furthermore, PsyCap not only mediates these relationships but also moderates them—buffering the detrimental effect of SA while enhancing the protective effect of NE. These insights suggest the value of a dual-focused approach to counselor support, combining training in adaptive emotional regulation with PsyCap development initiatives. Such integrated efforts may help mitigate burnout and enhance counselors’ professional effectiveness. Future research should employ longitudinal designs to better understand the temporal dynamics of these relationships and to explore boundary conditions, particularly the non-significant moderating role of PsyCap in the DA-JB relationship.

## Data Availability

The original contributions presented in this study are included in this article/Supplementary material, further inquiries can be directed to the corresponding author.
